# Association of statin use and increase in lipoprotein(a): a real-world database research

**DOI:** 10.1186/s40001-023-01155-x

**Published:** 2023-07-01

**Authors:** Tienan Feng, Yao Li, Xiongfeng Xue, Wei Yang, Qiang Li, Yushi Huang, Tengteng Zhu, Jue Wang, Limin Xu, Xianchen Li, Jing Gao, Shiming Sun, Bin Zhu, ShuYu Zhang, Beibei Cao, Jianwei Xuan, Zhigang Zhao, Biyun Qian

**Affiliations:** 1 Hongqiao International Institute of Medicine, Shanghai Tongren Hospital and School of Public Health, Shanghai Jiao Tong University School of Medicine, Shanghai, China; 2 Clinical Center for Intelligent Rehabilitation Research, Shanghai YangZhi Rehabilitation Hospital (Shanghai Sunshine Rehabilitation Center), School of Medicine, Tongji University, Shanghai, China; 3 SuValue Health Ltd, Shanghai, China; 4 Department of Pharmaceutical Outcomes and Policy, College of Pharmacy, University of Florida, Gainesville, FL USA; 5 Tongren Hospital, Shanghai Jiao Tong University School of Medicine, Shanghai, China; 6 Shanghai General Hospital, Shanghai Jiao Tong University School of Medicine, Shanghai, China; 7 Beijing Tiantan Hospital, Capital Medical University, Beijing, China; 8 Second Affiliated Hospital of Chengdu Medical College, China National Nuclear Corporation 416 Hospital, Chengdu, Sichuan China; 9 Department of Printing Equipment Engineering, Shanghai Publishing and Printing College, Shanghai, China

**Keywords:** Statin, Lipoprotein(a), Dyslipidemia, A real-world database

## Abstract

**Background:**

There is an increased concern that statins may have an unintended effect of elevated lipoprotein(a) [Lp(a)]. We conducted a large sample real-world study to test the association.

**Methods:**

This retrospective cohort study was conducted using data from an integrated SuValue database, which includes 221 hospitals across China covering more than 200,000 of population with longitudinal follow-up to 10 years. Propensity score matching was applied to identify two comparable cohorts with statin users and non-statin users. Detailed follow-up information such as Lp(a) levels were extracted. The hazard ratio was calculated on Lp(a) changes based on the statin usage cohorts. Detailed subgroup and different characteristic cohorts’ analyses were also conducted.

**Results:**

After baseline propensity score matching, a total of 42,166 patients were included in a 1:1 matched ratio between statin users and non-statin users. In the case of no difference in low density lipoprotein (LDL-C), Lp(a) was increased significantly with the use of statins (adjusted HR 1.47; 95% confidence interval [CI] 1.43–1.50). Lp(a) increase was observed in various subgroup analyses and different cohorts. The dose intensity of statin was positively associated with the evaluated Lp(a) level.

**Conclusion:**

The use of statins was associated with an increased risk of Lp(a) elevation compared with non-statin use counterparts. The clinical relevance of these increases needs to be addressed in surrogate marker trials and/or large, cardiovascular outcomes trials.

**Supplementary Information:**

The online version contains supplementary material available at 10.1186/s40001-023-01155-x.

## Introduction

Literature has demonstrated that the use of statins is associated with decreased mortality in people with high low-density lipoprotein (LDL-C) [1]. Statins lower LDL-C levels through inhibiting 3-hydroxy-3-methylglutaryl-coenzyme A (HMG-CoA) reductase, a key enzyme in the synthesis of cholesterol [2]. Several large controlled clinical trials have confirmed significant reductions in rates of coronary heart disease morbidity and death with long-term statin therapy in patients with a high level of LDL-C [3]. All statins appear to be effective in the reduction of cholesterol regardless of the type of statins or their potency [4, 5]. Although numerous studies have demonstrated the primary and secondary prevention benefits of statin use, recent studies have reported cardiovascular events still occur in these patients despite statin treatment and certain patients remain at significant cardiovascular risk even with intensive statin therapy [6, 7]. Although statins can control the elevation of LDL-C, the use of statins is potentially associated with increased levels of lipoprotein-a [Lp(a)] [8, 9], which have been implicated as an independent risk factor for MACE (MACE: cardiovascular death, myocardial infarction, stroke, coronary revascularization, or hospitalization for unstable angina) [4–7]. This association has subsequently been reinforced by epidemiological studies, meta-analyses, and Mendelian randomization studies [10, 11]. In 2018 and 2019, two separate meta-analyses concluded that Lp(a) was positively associated with statin use [8, 9]. These two studies hypothesized that statin use may increase Lp(a) while reducing LDL-C, and subsequently indirectly increase the risk of cardiovascular disease (CVD) due to elevated Lp(a). There are also studies showing no effect so it needs to be reiterated that the literature is not conclusive on this subject [12–14]. However, this hypothesis requires a larger sample size than current studies to be tested and study. Given the extensive use of statins in the Chinese population, it is critical to conduct a large sample real-world study to test the association of statin use and elevated Lp(a) level.

## Methods

### Data sources

Research data were extracted from SuValue database, which included 221 hospitals from 23 provinces, municipalities, or autonomous regions across China. The 221 hospitals included more than 200,000 patients from 176 general hospitals, 28 traditional Chinese Medicine hospitals, 14 maternal and childcare hospitals, and 3 specialized hospitals. Clinical data have been anonymized, standardized, and quality controlled before being imported into SuValue database. Patients’ longitudinal personal level data were extracted from the database. Due to the retrospective and non-interventional nature of the study and lack of individual patient identifiable information, no patient informed consent or ethical review is required per local policy on the use of electronic health data.

### Study population

We then excluded patients who only had one ambulatory/hospitalization visit, had less than six months of continuous coverage, had only one Lp(a) test, or had CVD at first entry (FE) when he/she visit the hospital at the first time. To control the selection bias of Lp(a) measurements. Lp(a) level of the patient should be measured in the same hospital with the same Lp(a) testing method. The critical difference of Lp(a) levels is to define increases or decreases beyond analytical variation in a given individual as 2 times the square root of 2 multiplied with the coefficient of variation (CV). The CVD included cardiovascular and cerebrovascular diseases, cerebral hemorrhage, cerebral infarction, myocardial infarction, coronary heart disease, atherosclerosis, and transient ischemic attack. Patients were subsequently divided into statin users and non-statin users. To make sure the statin use and non-statin use groups were comparable, we conducted propensity score matching with age, sex, comorbidity history which included diabetes/glycuresis, hypertension, and arteriosclerosis/vascular sclerosis, and duration of follow-up time. Lp(a), LDL-C level at FE, high-density lipoprotein (HDL-C), apolipoprotein(a) [APO-A], apolipoprotein(b) [APO-B], total cholesterol (TC), Triglyceride (TG), and C-reactive protein (CRP) were used to balance the baseline between statin use and non-statin use groups. The matched cohorts formed the Primary Study Cohort. The median time from the initial cohort entry to the last visit to the hospital was 3 years. The maximum follow-up was 10 years.

### Exposure assessment

Patients with statin use recorded at any time over the follow-ups in their health care data were defined as statin use group while patients who had no any statin use in their health care data and in the same time window with the patients of the statin use group were defined as non-statin use group. The dose intensity as an instrumental variable was a grade variable, which was calculated based on the dosage of statin-based drugs and the follow-up time using unsupervised classification methods. Patients in different groups correspond to different levels of drug exposure intensity. The association between statin use dose intensification, Lp(a) level, LDL-C level, and change were analyzed using linear regression modeling or conventional proportional hazard model. To analyze the different effects of a single statin, we excluded those patients who used more than one statin drug over the follow-up. Then, we compared the hazard ratio (HR) of Lp(a) elevation among different statin-based drugs using the conventional proportional hazard model.

### Baseline balance

Baseline patient characteristics were compared for both statin use and non-statin use groups for the following variables: age, gender, comorbidity history, duration of follow-up, Lp(a) at the first study entry, mean LDL-C, HDL-C, APO-A, APO-B, TC, TG, CRP level using R tools CMatching (Version 2^.^3^.^0) [15]. Lp(a) and LDL-C were continuous variables. Age, sex, comorbidity history, and duration of follow-up were classified as categorical variables. The index date for the study was the time when the initial Lp(a) was tested. The follow-up time was defined as from the index date to the time when the last measurement of Lp(a) was available. Age was broken down into three brackets, < 45, 45 to 65, and > 65. Comorbidity history had two categories, i.e., non-comorbidity or comorbidity. Comorbidity included diabetes/glycuresis, hypertension, and arteriosclerosis/vascular sclerosis. The duration of follow-up was divided into three-time windows, 0^.^5 to < 3 years, 3 to 5 years, and > 5 years.

### Statistical analysis

The primary outcome is the change of Lp(a) levels over time estimated by the linear fitting model in each group, statin use, and non-statin use. The critical difference of Lp(a) is defined as 2 times the square root of 2 multiplied with the CV. The dependent variable was the continue value of Lp(a) of different time points of one patient and the independent variables were the time point when the Lp(a) was tested. Age at the time point when the Lp(a) was tested was the adjusted variable. If the direction of the fitting line ascents from the first entry time to the last follow-up time, the level of Lp(a) increased. Otherwise, it did not increase. We also set another standard that the change of Lp(a) was calculated using linear modeling. The outcome is Lp(a) decrease or Lp(a) increase if the increased level of Lp(a) increases 50 mg/dl [16]. We compared the changes of Lp(a) between statin use and non-statin use patients in the primary cohorts using a conventional proportional hazard model to estimate hazard ratios and their 95% confidence intervals for the change of the Lp(a) levels. In addition, we adjusted our models for these additional potential confounders including sex, age, LDL-C, comorbidity history, HDL-C, APO-C, APO-B, and CRP. In secondary analyses, we conducted subgroup analysis stratified by comorbidity history, LDL-C level, Lp(a) level at the first entry, follow-up time, sex, and age. To assess the robustness of our results, we conducted several subgroup analyses in terms of age, sex, comorbidity history, mean LDL-C level from the first entry to the last visit, Lp(a) at first entry, the outcome of LDL-C. We also constructed three different cohorts to evaluate the robustness of the results from the Primary Study Cohort. A sensitivity analysis was also conducted to evaluate the efficacy of statin on Lp(a) change. All the statistical analyses were performed using R (version 4^.^0^.^5, R Project for Statistical Computing) (See Additional file 1 for Detailed description of statistical methods).

## Results

### Study population

**Fig. 1:**
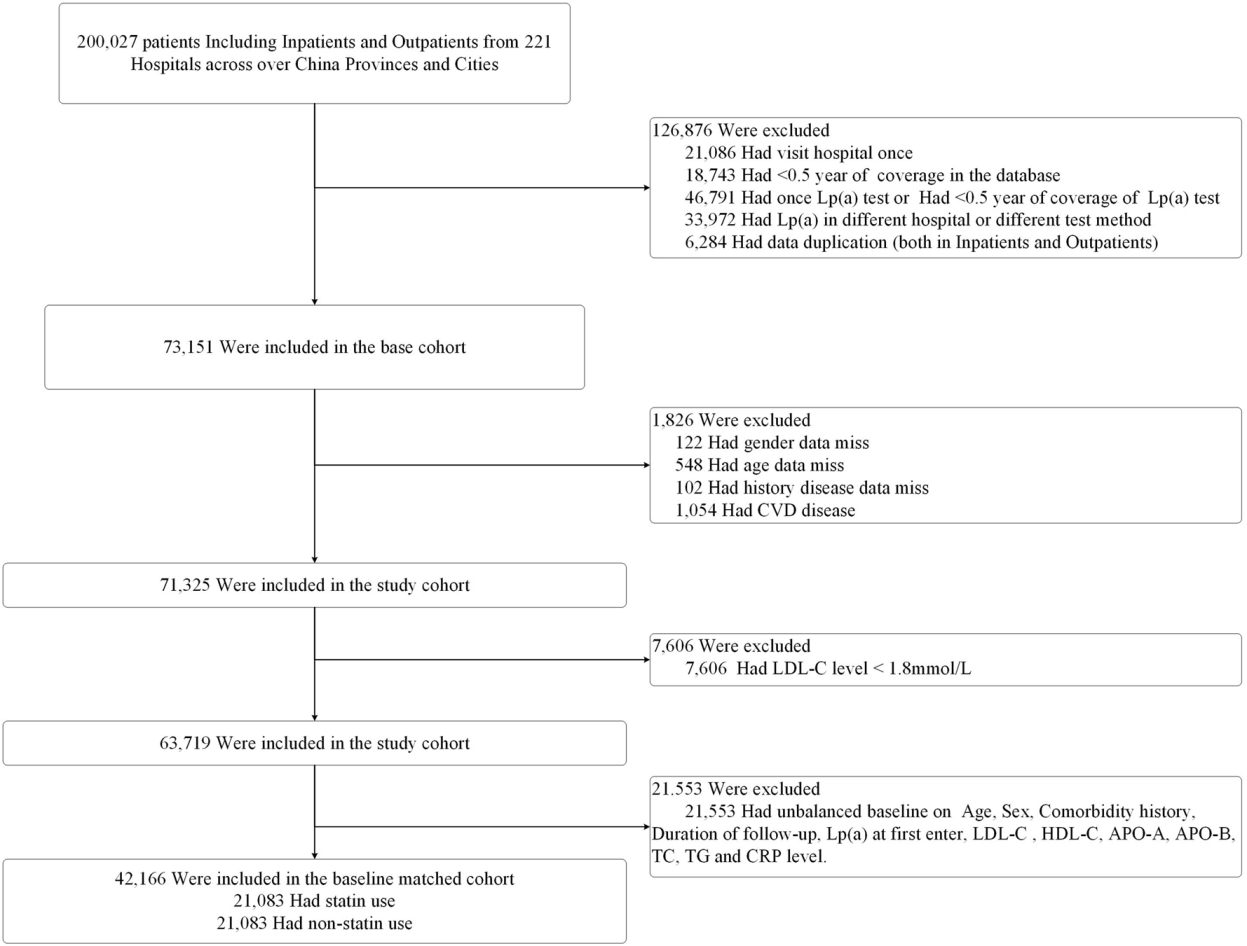
Numbers of patients in the Primary Study Cohort

A total of 200,027 patients at first entry were included from the database. After removing patients with one visit, continuous coverage less than 6 months, only one Lp(a) test, or < 0^.^5 year of coverage of Lp(a) test repeated data (both inpatients and outpatients) and whose Lp(a) were not measured in the same hospital with the same Lp(a) testing method, we constructed the base cohort, which included 73,151 patients. After deleting missing data and patients with CVD diagnosis at the first study entry, we derived the study cohort, which was composed of 71,325 patients. The final Primary Study Cohort included 42,166 patients after the Propensity Score Matching (PSM) for covariable balancing in a 1:1 ratio of the statin-used and non-statin used group (Fig. 1). In the Primary Study Cohort, 37,476 (88^.^88%)‬ patients were older than 45 years. In addition to hyperlipidemia, 33,525 (79^.^51%) patients had no comorbidity history while 8,641 (20^.^49%) had comorbidity history, including diabetes, hypertension, and arteriosclerosis (Additional file 2: Table S1). 39,073(92^.^66%) patients had medical encounters in the follow-up time window from 0^.^5 to 5 years. Lp(a) level at the first study entry, mean LDL-C, HDL-C, APO-A, APO-B, TC, TG, CRP level between the two groups was not significantly different (Table 1). The result of the three different cohorts to evaluate the robustness of the results from the Primary Study Cohort is also consistent (See Additional file 3 for Description of Other Three Cohorts).

**Table 1:** Baseline characteristics of patients with statin use and matched controls

Characteristic	Item	Non-statin use (*n* = 21,083)	Statin use (*n* = 21,083)
Age	< 45	2345(11.12%)	2345(11.12%)
	46–65	9947(47.18%)	9947(47.18%)
	> 65	8791(41.70%)	8791(41.70%)
Sex	Male	11,026(52.30%)	11,019(52.26%)
	Female	10,057(47.70%)	10,064(47.74%)
Comorbidity history^*^	Non-Comorbidity (FE)^#^	16,778(79.58%)	16,747(79.43%)
	Comorbidity (FE)	4305(20.42%)	4336(20.57%)
Follow-up time	[0^.^5–3) years	15,385(72.97%)	15,374(72.92%)
	[3–5) years	4154(19.70%)	4160(19.73%)
	≥ 5 years	1544(7.32%)	1549(7.35%)
Laboratory results	Lp(a)^$^ at FT (Mean, CI95%, mg/L)	7.99(4.07, 9.92)	7.98(3.88, 9.91)
	LDL-C (Mean, CI95%, mmol/L)	3.00(1.90, 4.63)	3.04(1.89, 4.91)
	HDL-C (Mean, CI95%, mmol/L)	1.30(0.74, 2.08)	1.30(0.76, 2.10)
	APO-A (Mean, CI95%, mmol/L)	1.33(0.83, 1.93)	1.33(0.86, 1.94)
	APO-B (Mean, CI95%, mmol/L)	0.99(0.58, 1.56)	0.99(0.58, 1.63)
	TC (Mean, CI95%, mmol/L)	5.06(3.41, 7.11)	5.11(3.43, 7.51)
	TG (Mean, CI95%, mmol/L)	1.63(0.58, 4.38)	1.68(0.60, 4.81)
	CRP (Mean, CI95%, mmol/L)	14.01(0.21, 85.20)	13.35(0.22, 76.50)

^*^Comorbidity: diabetes/glycuresis, hypertension, arteriosclerosis/vascular sclerosis

^#^FE: First entry time

^$^Lp(a): The variable was the log2 transform of Lp(a) level

### Association of statin-based drugs with change in lp(a) level

The conventional proportional hazard model analysis indicated that statin use, in comparison with non-statin use, was associated with an increased level of Lp(a) (hazard ratio = 1^.^43, and the 95% confidence interval: 1^.^40 to 1^.^47). Upon adjusting for age, LDL-C, comorbidity history, HDL-C, APO-C, APO-B, and the change of LDL-C, the association remained unchanged (hazard ratio = 1^.^47 and the 95% confidence interval: 1^.^43 to 1^.^50) (Table 2 and Additional file 2: Table S2). While the increased level of Lp(a) increases 50 mg/dl, the outcome of Lp(a) increases. Otherwise, the outcome of Lp(a) decreases. Then the adjusted association between statin use and Lp(a) level remained unchanged (hazard ratio = 1^.^40 and the 95% confidence interval: 1^.^36 to 1^.^43) (Table 2 and Additional file 2: Figure S1). Waterfall plots display the entire range of changes in Lp(a) levels in both the statin and non-statin use groups (Additional file 2: Figure S2). The graphs show significant variation in changes in Lp(a) in both groups. On average, statin use was associated with an increase of Lp(a) at 138^.^70 (95% Confidential Interval: − 174^.^00 to 682^.^90) mg/dl, and in the non-statin use group was 22^.^20 (95% Confidential Interval: − 387.40 to 457^.^90) mg/dl (Additional file 2: eFigure 2). A sensitivity analysis was also conducted. Results show that the effect of statin on Lp(a) elevation is robust (Additional file 2: Figure S3).

**Table 2:** Association between treatment with statin-based drugs versus non-statin use and the change of the Lp(a)

Item	Non-statin use (*n* = 21,083)	Statin use (*n* = 21,083)	HR(CI95%)	
			Model 1^*^	Model 2^@^
Primary outcome				
Lp(a) decrease (FU^#^)	8868(42.06%)	2956(14.02%)	1.43(1.40, 1.47)^&^	1.47(1.43, 1.50)^&^
Lp(a) increase (FU^#^)	12,215(57.94%)	18,127(85.98%)		
Lp(a) decrease (FU^$^)	14,204(67.37%)	8477(40.21%)	1.77(1.72, 1.82)^&^	1.40(1.36, 1.43)^&^
Lp(a) increase (FU^$^)	6879(32.63%)	12,606(59.79)		

^*^Changes in Lp(a) between statin use and non-statin use patients were compared using a conventional proportional hazard model

^@^Adjusted the proportional hazard model by Age, CRP, Follow-up(months), LDL-C, Comorbidity (FE), HDL-C, APO-A, APO-B,and the change of LDL-C

^#^The change of Lp(a) was calculated using linear mixed modeling. The outcome is Lp(a) decrease or Lp(a) increase. FU: Follow-up

^$^The change of Lp(a) was calculated using linear mixed modeling. The outcome is Lp(a) decrease or Lp(a) increase if the increased level of Lp(a) increases 50 mg/L. FU: Follow-up

^&^*p* < 0.05

### Subgroup analysis

**Fig. 2:**
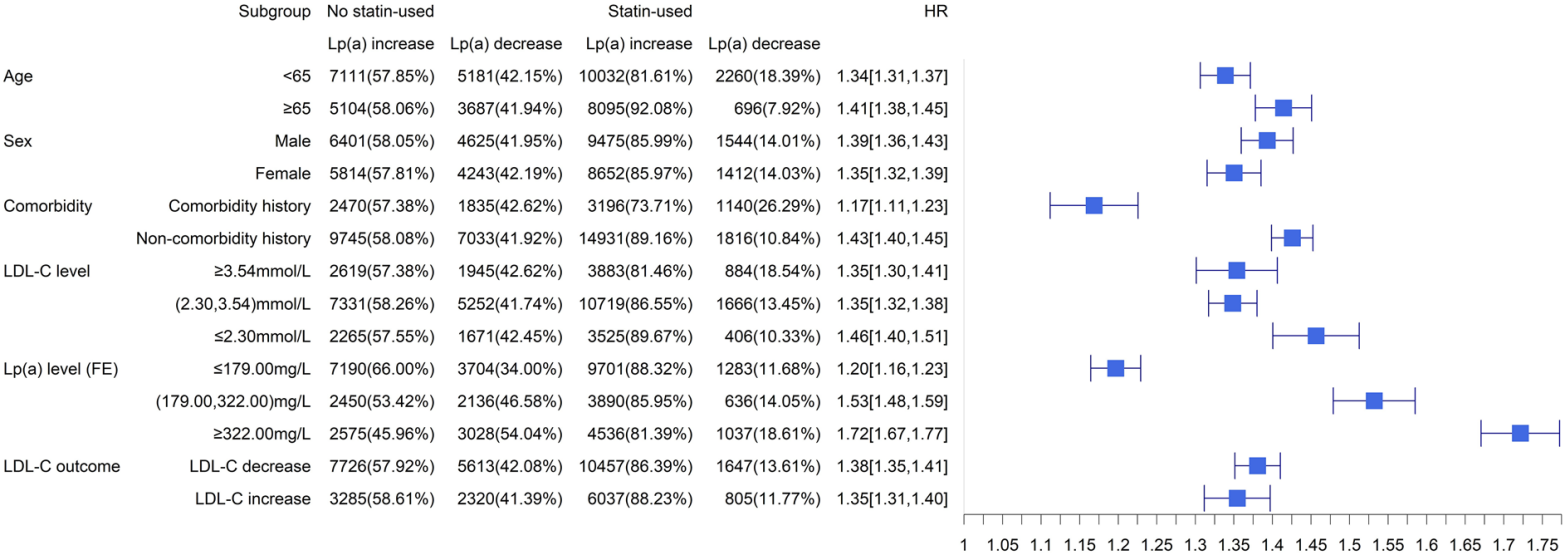
Subgroup and sensitive analysis: association between treatment with statin-based drugs and the change of Lp(a) of different subgroups of the Primary Study Cohort

Overall, the results of our subgroup analyses were consistent with those based on the Primary Study Cohort. In Primary Study Cohort, statin use in patients without comorbidity history had a higher risk of Lp(a) elevation than one with comorbidity, HR: 1^.^43, 95% CI 1^.^40 to 1^.^45 vs HR: 1^.^17, 95% CI 1^.^11 to 1.23. Patients 65 years of age or older had a higher risk of Lp(a) elevation than those younger than 65, HR: 1^.^41, 95% CI 1^.^38 to 1^.^45 vs HR: 1^.^34, 95% CI 1^.^31 to 1^.^37. The HRs of patients whose Lp(a) at the FE ≥ 322^.^00 mg/dl and ≤ 179^.^00 mg/dl were 1^.^72 (95% CI 1^.^67 to 1^.^77) and 1^.^20 (95% CI 1^.^16 to 1^.^23), respectively (Fig. 2).

### Exploratory analysis of association of lp(a) elevation with statin dose intensity and type of statin

**Fig. 3:**
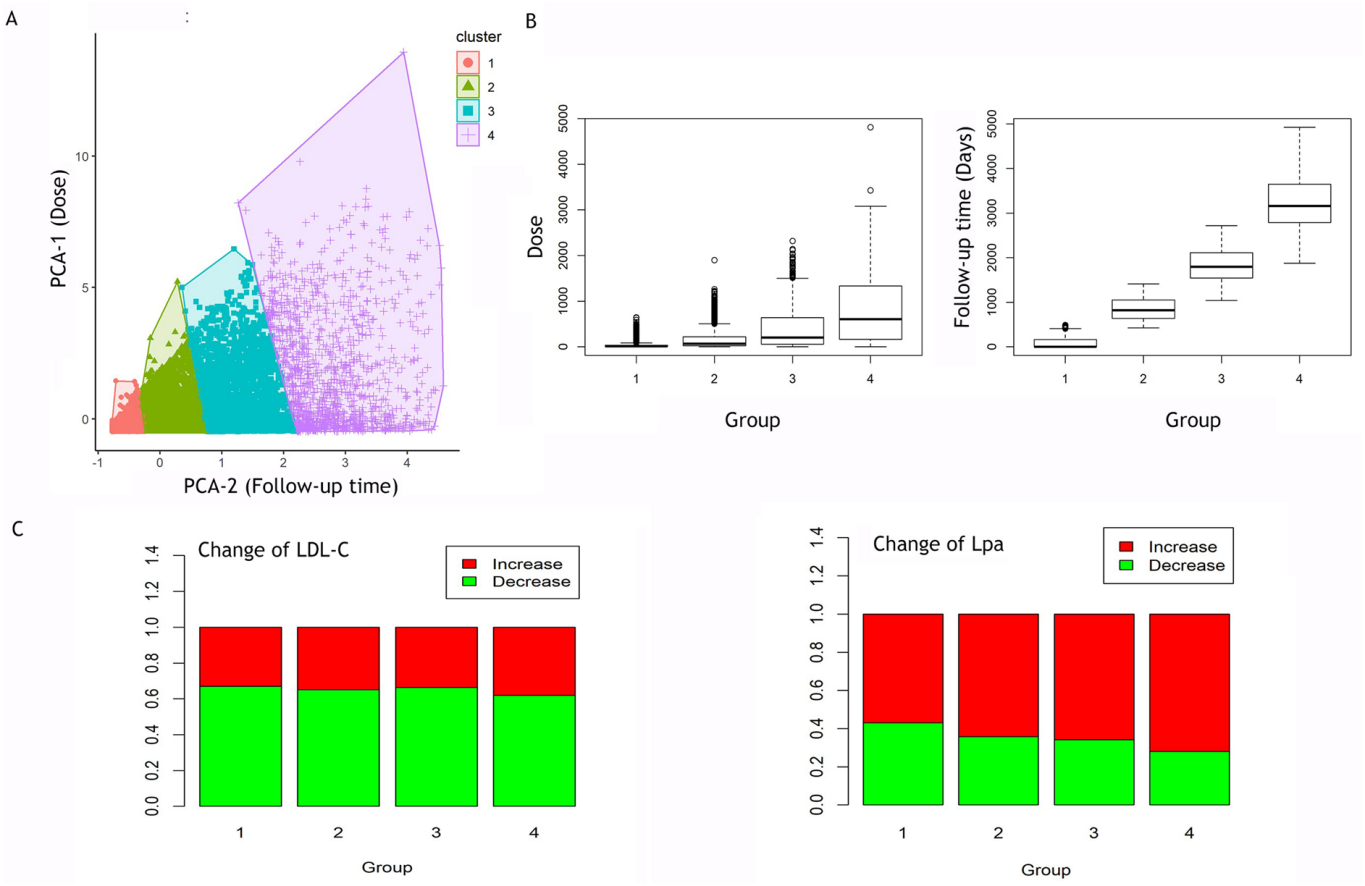
Association of Lp(a) elevation with statin intensity

We conducted an additional analysis of the association of Lp(a) increase with statin intensity using the Primary Study Cohort. According to the dose for statin-based drugs and follow-up time, patients with statin use were divided into four groups with different dose intensity in ascending order (Fig. 3A). Group 1 was the lowest drug intensity while group 4 was the highest intensity (Additional file 2: Table S3 and Fig. 3B). The dose intensity was associated with an increased level of Lp(a) (*p* < 0^.^01) and a stable level of LDL-C (*p* > 0^.^05) (Additional file 2: Table S4, Table S5, and Fig. 3C). High-intensity statin helped to control the elevation of LDL-C but increase the level of Lp(a). After excluding those patients who used more than one statin drug over the follow-up, statins used alone included Atorvastatin, Fluvastatin, Lovastatin, Pravastatin, and Simvastatin, Atorvastatin was used mostly while Lovastatin was used in the shortest time (Additional file 2: Table S6, Table S7, and Figure S4). We set the Atorvastatin as the reference. The risk of Lp(a) elevation of Fluvastatin and Simvastatin was less than Atorvastatin while Pravastatin had a higher risk of Lp(a) elevation (Additional file 2: Table S8).

## Discussion

This large-scale real-world study found that the use of statin drugs was associated with an increased risk of Lp(a) elevation based on a real-world study setting and this association was observed over multiple patient cohorts constructed with different criteria. A previous study reported that patients receiving moderate- to high-intensity statins were still been observed with CVD events in the follow-up period [17, 18]. It has been reported that elevated Lp(a) levels may be associated with an increase in the thickness of atherosclerotic plaques, but this hypothesis remains to be confirmed [19]. To find the reason why certain patients remain at significant cardiovascular risk even with statin therapy, many biochemical indices were researched. Recent two meta-analysis [8, 9] reported that statin use increased the Lp(a) level; and Lp(a) had been implicated as an independent risk factor of CVD [20–23], while other two papers that did not show an association between Lp(a) and statin [12, 13]. Currently, many articles analyzing this relationship are based on meta-analyses in which the included studies primarily focus on other endpoints rather than the association between Lp(a) and statin, resulting in some bias in the conclusions. Therefore, more studies with larger sample sizes using statins and increasing Lp(a) levels as the primary endpoint are needed to obtain stronger evidence, especially in the real world [24, 25]. To address this association, we conducted this real-world study with more than 70,000 patients which is the largest sample size to address the issue. The median time for the follow-up was 3 years. 10 years were the maximum. In our real-world study, the results of the Primary Study Cohort revealed that statin use was associated with Lp(a) elevation. Results of the Primary Study Cohort have shown statin use was associated with a mean increase of Lp(a) by 138.70 mg/dl with its 95% confidence interval ranging from − 17.40 to 682.90 mg/dl. It was reported that Lp(a) (< 30 mg/dl) is associated with a decreased risk of cardiovascular disease such as peripheral vascular disease, stroke, heart failure, and aortic stenosis [16, 26]. On the contrary, in patients who did not use a statin, a mean increase of Lp(a) by 222^.^00 mg/dl was observed with its 95% confidence interval ranging from − 387^.^40 to 457^.^90 mg/dl. Similar findings were noticed from various patients’ subgroup cohorts. Although our study was observational in nature and thus subject to potential confounding, we used rigorous matching and statistical adjustment to minimize confounding effects.

In addition to the analysis from multiple cohorts constructed with different criteria, we conducted several subgroup analyses with respect to age, gender, comorbidity disease, follow-up time, average Lp(a) level, average LDL-C level, and changes in LDL-C value with the use of statin. There was little research reporting the risk of age, comorbidity history, and Lp(a) level at the first entry for Lp(a) elevation when patients use a statin. Based on the result of the Primary Study Cohort, the degree of association of statin use with Lp(a) varied among different subgroups. Patients who were older than 65, with comorbidity history, or high Lp(a) level at first entry had a higher risk of Lp(a) elevation when they used a statin.

To further explore the potential role of dose and category of statin, we formed an instrumental variable set as drug intensity, for the diversity of prescription dosage and statin used time. M de Boer et al. reported that none of the types of statins changed Lp(a) significantly compared to placebo (very low- to high-certainty evidence), as well as intensities of statin therapy (low- to moderate-certainty evidence) [12]. Our result from the Primary Study Cohort, used an instrumental variable and provided the evidence to support that the level of Lp(a) was positively associated with statin intensity while the level of LDL-C was not associated with statin intensity, which was consistent with the previous study [3, 27–29]. The analysis excluded those patients who used more than one statin drug over the follow-up and set the most widely used statin, Atorvastatin, as a reference. The results revealed Pravastatin had a higher risk of Lp(a) elevation while Simvastatin and Fluvastatin had a relatively lower risk.

The real-world study faces many data-quality-related challenges. There were a number of important variables that were not collected but were associated with the change of Lp(a), such as lifestyle, diet, BMI, smoke status, and concomitant drug use [30]. Then, no information on statin treatment continuity is provided, although patients with only one statin treatment were excluded. In addition, the drug dosage as recorded in the database may not be accurate and the follow-up times of different patients varied. We only had the prescription dosage and days of supply. It was unknown whether the patients actually took the medicines as prescribed. We also found the prescription dose strength and administration schedules varied for the same statin. These factors might bring errors or biases.

## Conclusion

In summary, we found that patients with statin use had a high risk of elevated Lp(a). This finding was supported by evidence from multiple cohorts constructed with different criteria and all were balanced with respect to known factors which may affect the change of Lp(a). This longitudinal patient-level follow-up real-world study indicated that the use of statin drugs was associated with an increased risk of Lp(a) elevation which might potentially lead to increased CVD events. This finding was consistent in separate cohorts and subgroup analyses for patients with different characteristics. To better manage patients based on the characteristics of Lp(a). The measurement of Lp(a) should be standardized. Our findings could serve as the basis for subsequent real-world studies investigating the relationship between the increase of Lp(a) levels and augmented CVD risk. Randomized controlled trials to see if targeted lowering of Lp(a) improves clinical outcomes should be conducted.

## Supplementary Information

**Additional file 1:** Detailed description of statistical methods.

**Additional file 2: Figure S1.** Detailed description of eFigures and eTables.

**Additional file 3: **Description of other three cohorts.

## Data Availability

The datasets used and/or analyzed during the current study are available from the corresponding author on reasonable request.

## Abbreviations

Lp(a): Lipoprotein(a)
LDL-C: Low-density lipoprotein
HR: Hazard ratio
CI: Confidence interval
AHMG-CoA: 3-hydroxy-3-methylglutaryl-coenzyme
CVD: Cardiovascular disease
CV: Coefficient of variation
HDL-C: High-density lipoprotein
APO-A: Apolipoprotein(a)
APO-B: Apolipoprotein(b)
TC: Total cholesterol
TG: Triglyceride
CRP: C-reactive protein
PSM: Propensity Score Matching
FE: First entry time
FU: Follow-up
