# Supplementary material for: Association of statin use and increase in lipoprotein(a): a real-world database research
Source: Eur J Med Res. 2023 Jul 1;28:212. doi: 10.1186/s40001-023-01155-x (PMC10314451; doi:10.1186/s40001-023-01155-x)
Supplement: Supplementary file 1 — Additional file 1: Detailed description of statistical methods. [file 40001_2023_1155_MOESM1_ESM.docx]

# **Detailed description of statistical methods**

Before analysis, our cases and controls were matched with age, sex, comorbidity history which including diabetes/glycuresis, hypertension, and arteriosclerosis/vascular sclerosis, and duration of follow-up time, Lp(a) at the first study entry and mean LDL-C level from the first entry (named as LDL-C for short as follow) to the last visit using R tools CMatching (Version 2.3.0)^1^. Lp(a) and LDL-C were continuous variables. Age, sex, comorbidity history and duration of follow-up were classified as categorical variables. The index date for the study was the time when the initial Lp(a) was tested. The follow-up time was defined as from the index date to the time when the last measurement of Lp(a) available. Age was broken down into three brackets, <45, 45 to 65, and >65. Comorbidity history had two categories, i.e., non-comorbidity or comorbidity. Comorbidity included diabetes/glycuresis, hypertension, and arteriosclerosis/vascular sclerosis. Duration of follow-up was divided into three-time windows, 0.5 to < 3 years, 3 to 5 years, and > 5 years.

Patients with statin-use recorded at any time over the follow-ups in their health care data were defined as statin use group while patients who had no any statin use in their health care data and in the same time window with the patients of statin use group were defined as non-statin use group. For the time points of Lp(a) test from patients were different, the change of Lp(a) levels over time was estimated by linear fitting modeling between patients of statin use and non-statin use. The dependent variable was the level of Lp(a) of different time points of one patient and the independent variables was the time point when the Lp(a) was tested. In addition to demography (age and gender) and comorbidity history, base-line information of apolipoprotein-A (APO-A), apolipoprotein-B (APO-B), C-reaction protein (CRP), LDL-C, high-density lipoprotein cholesterol (HDL-C), total cholesterol (TC), triglyceride (TG), were included in the model. The critical difference of Lp(a) is defined as 2 times the square root of 2 multiplied with the coefficient of variation (CV). We compared the change of Lp(a) between statin use and non-statin use patients in the primary cohorts using a conventional proportional hazard model to estimate hazard ratios and their 95% confidence intervals for the change of the Lp(a) levels. In addition, we adjusted our models for these additional potential confounders including sex, age, LDL-C, comorbidity history, HDL-C, APO-C, APO-B and CRP. we used three-quartile method to divided patients in three group, Lp(a) level ≤179 mg/L, Lp(a) from 179 to 322 mg/L and ≥ 322 mg/L. We set Lp(a) level ≤179 mg/L as the reference, and use a conventional proportional hazard model to assess the dose-response relationship between the risk of elevated Lp(a) levels.

To assess the robustness of our results, we conducted several a subgroup analyses in the term of age, sex, comorbidity history, mean LDL-C level from the first entry to the last visit, Lp(a) at first entry, the outcome of LDL-C. The included patients were divided by age (<65 or ≥ 65), sex (female or male), comorbidity history (have or not ), mean LDL-C level from the first entry to the last visit which were stratified into three group by three-quartile method, Lp(a) at first entry which were stratified into three group by three-quartile method and the outcome of LDL-C which was calculated using linear mixed modeling respectively. The change of Lp(a) in different subgroup between statin use and non-statin use patients in the primary cohorts using a conventional proportional hazard model to estimate the adjusted hazard ratios and their 95% confidence intervals for the change of the Lp(a) levels which was adjusted by sex, age, LDL-C, comorbidity history, HDL-C, APO-C, APO-B, CRP and the change of LDL-C. If one of the covariables was used as the stratification, the covariable did not used for adjusted. The subgroup analyses were conducted separately among patients with and without a history of statin use. We used conventional proportional hazard model to estimate HRs and corresponding 95% confidence intervals (CIs) for the association between statin-based drugs and the risk of Lp(a) elevation. Besides, we also conducted the sensitivity analysis of the robustness. The information regarding on factors that may influence the change of Lp(a) were unknown. We used the LDL-C and HDL-C are the benchmark covariates. For the unknown factors including diet and exercise habits were associated with the levels of LDL-C and HDL-C. These arguments parameterize how many times stronger the confounder is related to the statin exposure was set 3. Sensitivity contour plots of point estimates and t-values and extreme scenarios were used to evaluated the robustness.

We also constructed another three cohorts with different clinical characteristics to assess the robustness of our results, including all Study Cohort, in which all patients and the counterparts were included regardless whether the LDL-C level ≥ 1.8 mmol/L or not; LDL-C Stable Cohort, in which the LDL-C level of patients with statin was not elevated from first entry to the last visit. Normal LDL-C Cohort, in which the LDL-C level of patients was < 1.8mmol/L. The baseline of counterpart with non-statin use in all the cohorts was balanced with age, sex, comorbidity history, duration of follow-up, Lp(a) at first enter and the LDL-C level. All analyses were conducted separately among patients with and without a history of statin use. We used conventional proportional hazard model to estimate HRs and corresponding 95% confidence intervals (CIs) for the association between statin-based drugs and the risk of Lp(a) elevation (see the description other three cohorts section).

To assess the dose-efficacy relationship between statin, Lp(a) elevation and CVD events, an instrumental variable is constructed. The instrumental variable a grade variable, which was calculated based on dosage of statin-based drugs and the follow-up time using unsupervised classification methods, K-means^2^. Patients in different groups correspond to different levels of drug exposure intensity. The association between statin use dose intensify, Lp(a) level, and LDL-C level were analyzed using linear fitting modeling which were adjusted by age, sex, comorbidity (FE), HDL-C, APO-A, APO-B, Follow-up time. We used conventional proportional hazard model to estimate HRs and corresponding 95% CIs for the association between statin intensity, Lp(a) elevation. To analyze the difference effect of single statin, we excluded those patients who used more than one statin drug over the follow-up. Then, we compared the hazard ratio of Lp(a) elevation and CVD event among different statin-based drugs including Atorvastatin, Fluvastatin, Lovastatin, Pravastatin, Rosuvastatin and Simvastatin using conventional proportional hazard model which was adjusted by age, sex, comorbidity (FE), LDL-C, Lp(a) at FE. The used most statin drug, Atorvastatin, was set as the reference.

All the statistical analyses were performed using R (version 3.6.3, R Project for Statistical Computing).
