# Supplementary material for: Association of statin use and increase in lipoprotein(a): a real-world database research
Source: Eur J Med Res. 2023 Jul 1;28:212. doi: 10.1186/s40001-023-01155-x (PMC10314451; doi:10.1186/s40001-023-01155-x)
Supplement: Supplementary file 2 — Additional file 2: Figure S1. Detailed description of eFigures and eTables. [file 40001_2023_1155_MOESM2_ESM.docx]

**CONTENT**

[eFigure 1 Comparison of survival curves in patients with the change of the Lp(a) of statin-based drugs versus non-statin use: A. The Primary Study Cohort; B. The Cohort with balanced censor counts. 1](#_Toc21581)

[eFigure 2. Change intensity of Lp(a) between patients of statin use and non-statin use: A. Results of non-statin use patients; B. Results of statin use patients. 2](#_Toc15342)

[eFigure 3 Sensitivity analysis of statin effect on Lp(a) Change: A. Contour plots for the point estimate; B. Contour plots for the sensitivity of the t-value; C. Sensitivity plots of extreme scenarios. 3](#_Toc22928)

[eFigure 4. Distribution of statin drug. 4](#_Toc18955)

[eTable 1. Distribution of comorbidity history coexisting with hyperlipidemia. 5](#_Toc1958)

[eTable 2. Analysis between statin use and the Lp(a) elevation using conventional proportional hazard model. 6](#_Toc8222)

[eTable 3 Dose and follow-up time of different drug intensity. 7](#_Toc10121)

[eTable 4. Association between drug intensity and Lp(a) increase using multiple-factor linear regression analysis. 8](#_Toc4285)

[eTable 5. Association between drug intensity and LDL-C decrease using multiple-factor linear regression analysis. 9](#_Toc16627)

[eTable 6. Time coverage of different statin drug. 10](#_Toc28239)

[eTable 7. Outcome of Lp(a) change of different statin drug. 11](#_Toc26617)

[eTable 8. Results of different statin-based drug on change the Lp(A) level using conventional proportional hazard model. 12](#_Toc686)


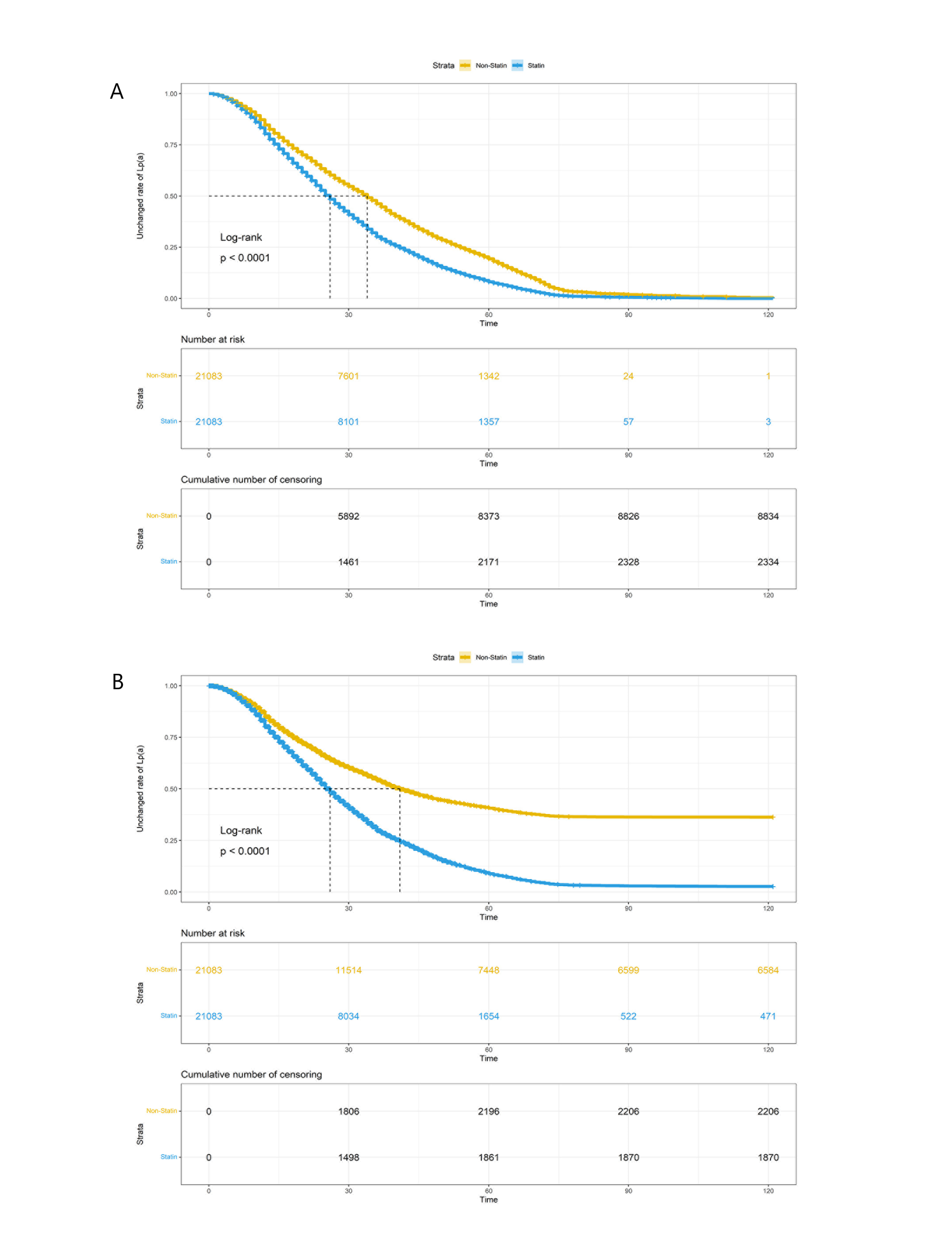


# eFigure 1 Comparison of survival curves in patients with the change of the Lp(a) of statin-based drugs versus non-statin use: A. The Primary Study Cohort; B. The Cohort with balanced censor counts.


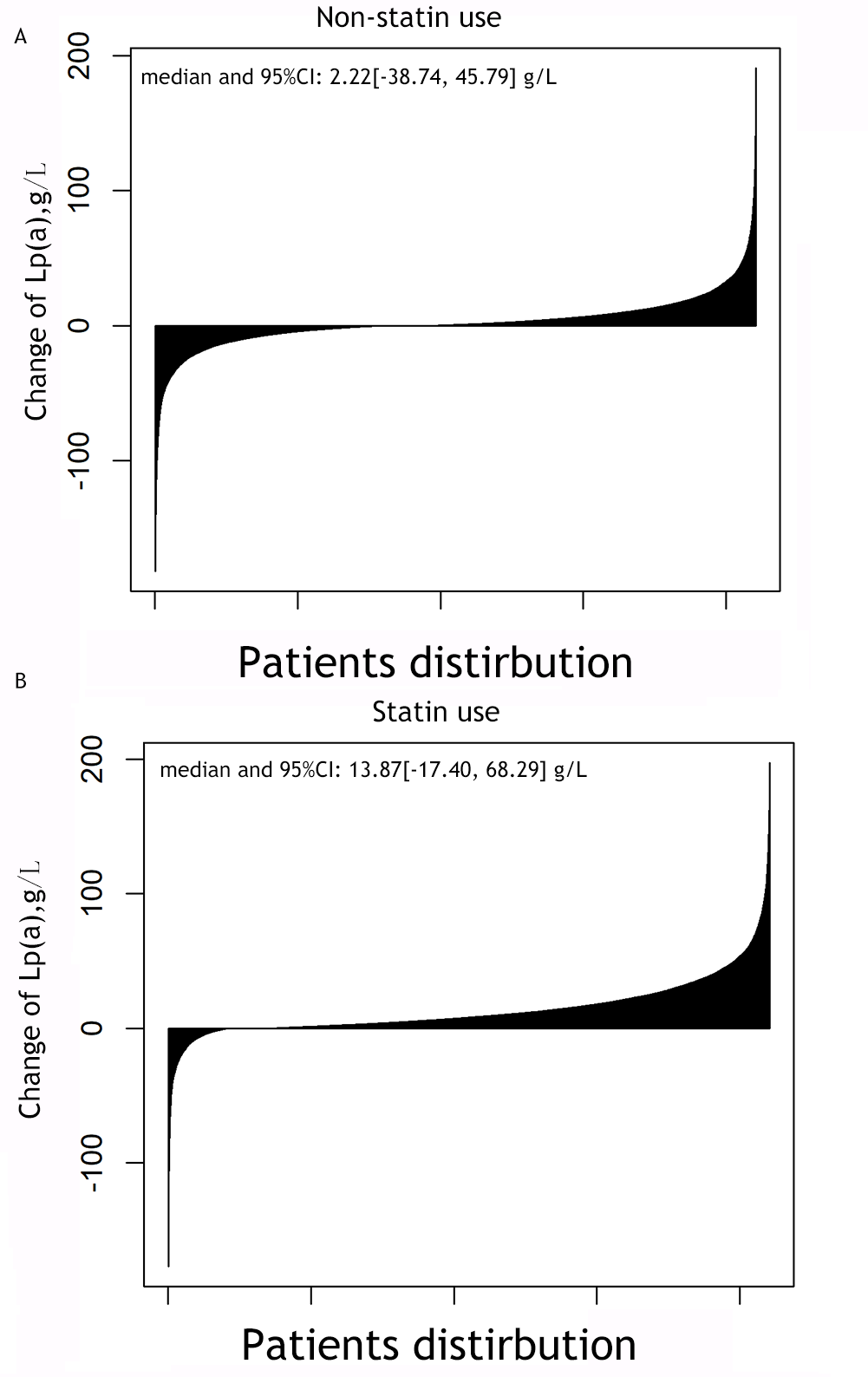


# eFigure 2. Change intensity of Lp(a) between patients of statin use and non-statin use: A. Results of non-statin use patients; B. Results of statin use patients.


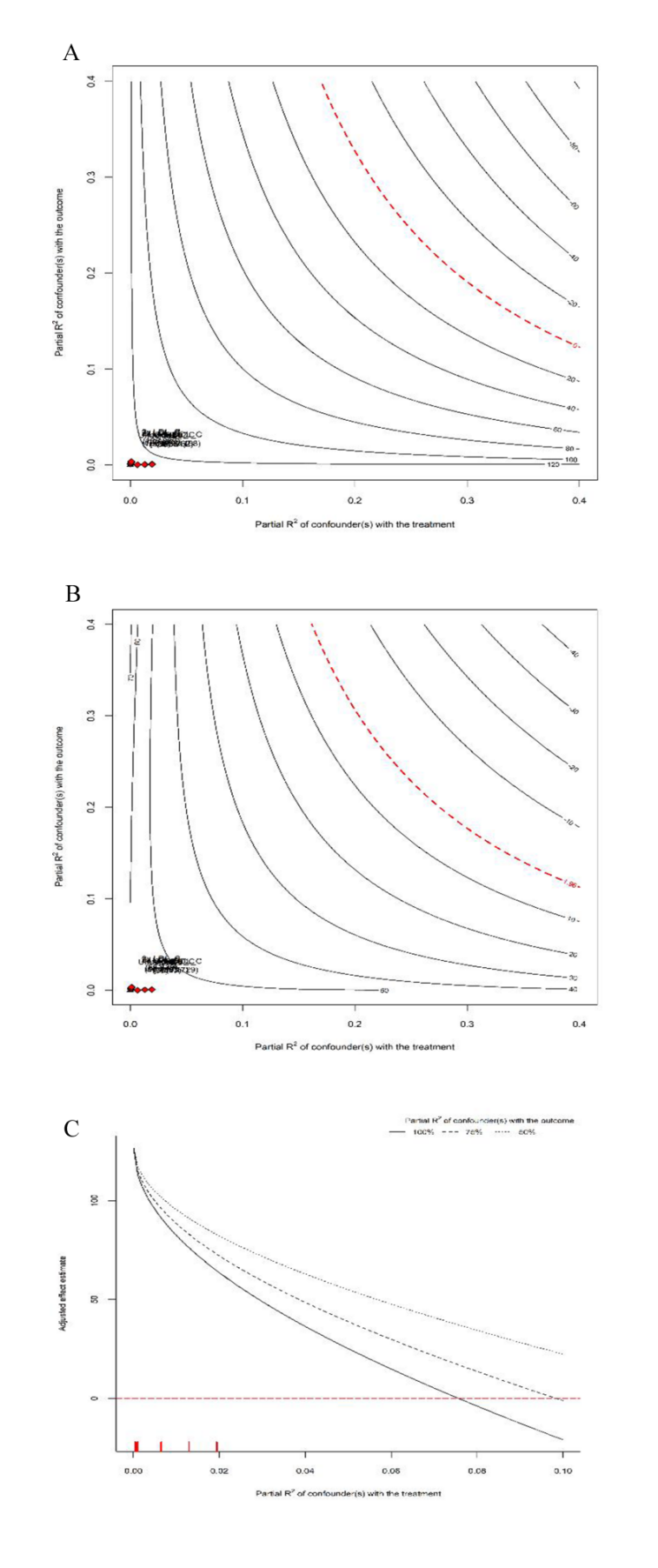


**eFigure 3 Sensitivity analysis of statin effect on Lp(a) Change:** A. Contour plots for the point estimate; B. Contour plots for the sensitivity of the t-value; C. Sensitivity plots of extreme scenarios.


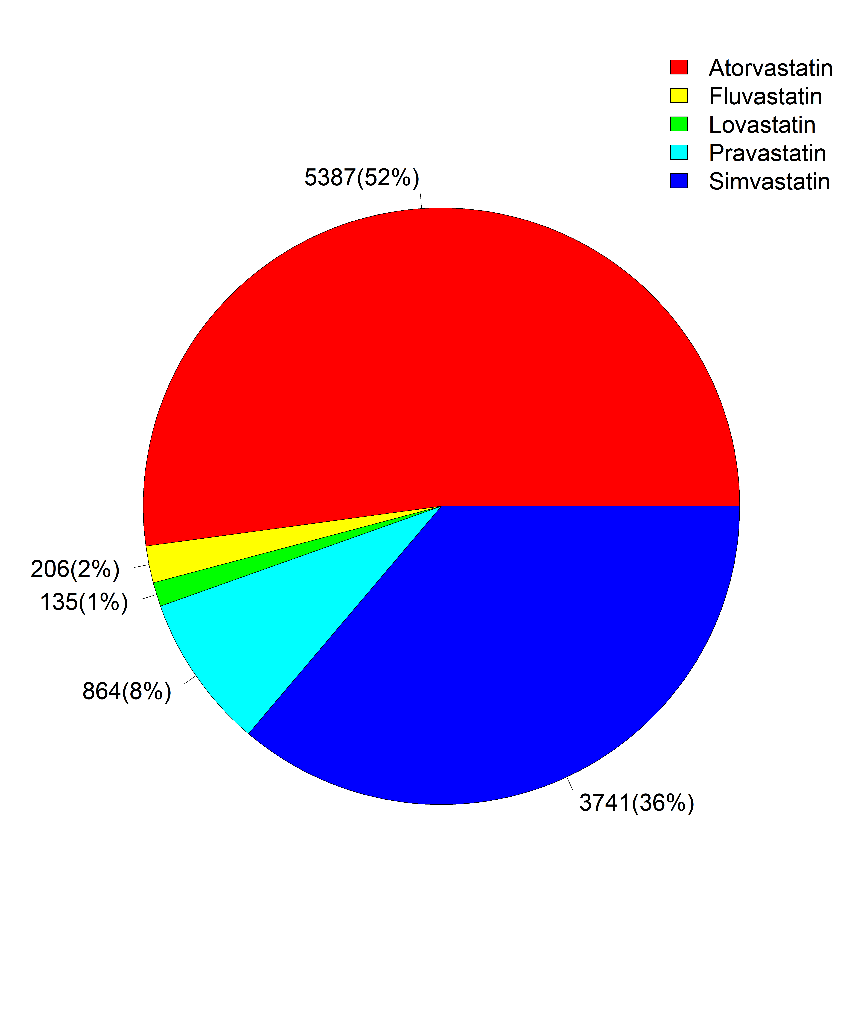


# eFigure 4. Distribution of statin drug.

# eTable 1. Distribution of comorbidity history coexisting with hyperlipidemia.

| **Comorbidity History** | **Non-statin use (n = 4305)** | **Statin use (n = 4336)** |
| --- | --- | --- |
| **Diabetes** | 2039 | 1732 |
| **Hypertension** | 1793 | 2020 |
| **Arteriosclerosis** | 128 | 97 |
| **Others** | 345 | 487 |

# eTable 2. Analysis between statin use and the Lp(a) elevation using conventional proportional hazard model.

|  | **HR** | **95%CI** | **Zvalue** | **Pvalue** |
| --- | --- | --- | --- | --- |
| **Sex** | 0.98 | (0.95,1.00) | -1.88 | 0.06 |
| **Age** | 1.25 | (1.23,1.27) | 21.58 | <0.01 |
| **Statin use** | 1.47 | (1.43,1.50) | 31.77 | <0.01 |
| **CRP** | 1 | (1.00,1.00) | 4.91 | <0.01 |
| **LDL-C** | 1.18 | (1.15,1.21) | 11.16 | <0.01 |
| **Comorbidity history (FE)** | 1.06 | (1.05,1.06) | 14.35 | <0.01 |
| **HDL-C** | 0.9 | (0.85,0.96) | -3.61 | <0.01 |
| **APO-A** | 0.87 | (0.80,0.95) | -3.61 | <0.01 |
| **APO-B** | 0.71 | (0.64,0.78) | -9.55 | <0.01 |
| **Change of LDL-C** | 0.93 | (0.91,0.95) | -7.17 | <0.01 |

^#^FE: First entry time.

# eTable 3 Dose and follow-up time of different drug intensity.

| **Group(counts)** | **Sum Cumulative dose**  **(95%CI, Unit: piece)** | **Follow-up time (95%CI, Unit: month)** |
| --- | --- | --- |
| **Group-1** | 35.41(1.00, 224.00) | 25.16(3.00, 70.65) |
| **Group-2** | 163.49(3.00, 768.00) | 28.38(5.00, 69.00) |
| **Group-3** | 390.76(5.00, 1417.40) | 38.19(7.00, 77.00) |
| **Group-4** | 814.94(22.00, 2501.32) | 39.34(7.00, 85.00) |

# eTable 4. Association between drug intensity and Lp(a) increase using multiple-factor linear regression analysis.

|  | **RC^*^** | **95%CI** | **Z value** | ***p* value** |
| --- | --- | --- | --- | --- |
| **Intercept** | -160.53 | (-207.34,-113.72) | -6.72 | <0·01 |
| **Group (drug intensity)** | 43.68 | (37.86,49.49) | 14.73 | <0·01 |
| **Age** | 3.64 | (3.21,4.07) | 16.58 | <0·01 |
| **LDL-C** | 44.57 | (35.41,53.72) | 9.55 | <0·01 |
| **Comorbidity (FE)^#^** | 4.9 | (1.68,8.13) | 2.98 | <0·01 |
| **HDL-C** | 35.56 | (11.66,59.47) | 2.92 | 0·36 |
| **APO-A** | -76.56 | (-107.07,-46.05) | -4.92 | 0·01 |
| **APO-B** | 111.17 | (84.45,137.90) | 8.15 | 0·03 |
| **Follow-up time** | 16.54 | (7.94,25.15) | 3.77 | 0·67 |
| **Sex** | 12.23 | (1.54,22.91) | 2.24 | 0·13 |

^*^Regression coefficients that were calculated by multiple linear regression model. The dependent variable was the log2 transform of Lp(a) level.

^#^FE: First entry time.

# eTable 5. Association between drug intensity and LDL-C decrease using multiple-factor linear regression analysis.

|  | **RC^*^** | **95%CI** | **Z value** | ***p* value** |
| --- | --- | --- | --- | --- |
| **Intercept** | 0.76 | 0.76(0.69,0.83) | 20.91 | <0·01 |
| **Group (drug intensity)** | 0.00 | (-0.01,0.00) | -0.97 | 0.33 |
| **Age** | 0.04 | (0.02,0.06) | -9.89 | <0·01 |
| **Comorbidity (FE) ^#^** | 0.02 | (0.02,0.03) | 9.26 | <0·01 |
| **HDL-C** | 0.27 | (0.24,0.31) | 14.62 | <0·01 |
| **APO-A** | -0.08 | (-0.13, -0.03) | -3.42 | <0·01 |
| **APO-B** | 2.08 | (2.05,2.11) | 141.42 | <0·01 |
| **Follow-up time** | -0.03 | (-0.04, -0.01) | -4.06 | <0·01 |
| **Sex** | 0.07 | (0.05,0.08) | 7.97 | <0·01 |

^*^Regression coefficients that were calculated by multiple linear regression model. The dependent variable was the log2 transform of Lp(a) level.

^#^FE: First entry time.

# eTable 6. Time coverage of different statin drug.

| **Drug Name** | **Start Time** | **End Time** |
| --- | --- | --- |
| **Atorvastatin** | 2006/2/23 | 2019/7/15 |
| **Fluvastatin** | 2005/12/29 | 2019/7/8 |
| **Lovastatin** | 2005/4/7 | 2010/1/18 |
| **Pravastatin** | 2006/2/7 | 2019/7/12 |
| **Simvastatin** | 2005/1/22 | 2019/7/12 |

# eTable 7. Outcome of Lp(a) change of different statin drug.

|  | **Lp(a) descend** | **Lp(a) elevation** |
| --- | --- | --- |
| **Atorvastatin** | 781 | 4606 |
| **Fluvastatin** | 42 | 164 |
| **Lovastatin** | 39 | 96 |
| **Pravastatin** | 86 | 778 |
| **Simvastatin** | 593 | 3148 |

# eTable 8. Results of different statin-based drug on change the Lp(A) level using conventional proportional hazard model.

|  | **HR** | **95%CI** | **Z value** | ***p* value** |
| --- | --- | --- | --- | --- |
| **Atorvastatin^*^** | 1.00 | - | - | - |
| **Fluvastatin** | 0.64 | (0.55,0.73) | -9.99 | <0.01 |
| **Lovastatin** | 1.20 | (1.02,1.38) | 2.03 | 0.04 |
| **Pravastatin** | 1.37 | (1.32,1.43) | 10.70 | <0.01 |
| **Rosuvastatin** | 0.88 | (0.85,0.91) | -7.48 | <0.01 |
| **Simvastatin** | 1.00 | (0.99,1.00) | -6.87 | <0.01 |
| **Age** | 0.98 | (0.96,1.00) | -2.17 | 0.03 |
| **LDL-C** | 1.00 | (1.00,1.00) | -14.93 | <0.01 |
| **Lp(a) at FE** | 1.09 | (1.08,1.10) | 15.84 | <0.01 |
| **Comorbidity history (FE) ^#^** | 0.98 | (0.95,1.01) | -1.48 | 0.14 |
| **Sex** | 0.64 | (0.55,0.73) | -9.99 | <0.01 |

^*^The reference drug.

^#^FE: First entry time.

**Table 1. Baseline characteristics of patients with statin use and controls of All Study Cohort ^$^.**

| **Characteristic** | **Item** | **Non-statin use (n=35753)** | **Statin use (n=35572)** |
| --- | --- | --- | --- |
| Age^&^ | <45 | 9492(26.55%) | 2707(7.61%) |
|  | 46-65 | 15917(44.52%) | 14306(40.22%) |
|  | >65 | 10344(28.93%) | 18559(52.17%) |
| Sex^&^ | Male | 18394(51.45%) | 17948(50.46%) |
|  | Female | 17359(48.55%) | 17624(49.54%) |
| Comorbidity history^*,&^ | Comorbidity history (FE)^#^ | 30870(86.34%) | 23892(67.17%) |
|  | Non- Comorbidity history (FE) | 4883(13.66%) | 11680(32.83%) |
| Follow-up time^&^ | [0·5 - 3) years | 27583(77.15%) | 23943(67.31%) |
|  | [3 - 5) years | 6129(17.14%) | 8192(23.03%) |
|  | ≥ 5 years | 2041(5.71%) | 3437(9.66%) |
| Laboratory results | Lp(a)^@^ at FE (Mean, CI95%, mg/L) ^&^ | 7.88(3.58, 9.87) | 7.81(3.19, 9.85) |
|  | LDL-C (Mean, CI95%, mmol/L) ^&^ | 2.78(1.24, 4.50) | 2.89(1.30, 4.87) |
|  | HDL-C (Mean, CI95%, mmol/L) ^&^ | 1.32(0.68, 2.14) | 1.27(0.72, 2.05) |
|  | APO-A (Mean, CI95%, mmol/L) ^&^ | 1.34(0.76, 2.00) | 1.31(0.81, 1.94) |
|  | APO-B (Mean, CI95%, mmol/L) ^&^ | 0.93(0.46, 1.50) | 0.96(0.50, 1.60) |
|  | TC (Mean, CI95%, mmol/L) ^&^ | 4.82(2.75, 6.99) | 4.97(2.91, 7.48) |
|  | TG (Mean, CI95%, mmol/L) ^&^ | 1.54(0.52, 4.41) | 1.77(0.60, 5.10) |
|  | CRP (Mean, CI95%, mmol/L) ^&^ | 13.49(0.17, 81.67) | 12.97(0.18, 76.93) |

^$^In the cohort (All Study Cohort), all patients and the counterparts were included.

^*^Comorbidity history: diabetes/glycuresis, hypertension, arteriosclerosis/vascular sclerosis, besides high blood lipids/hyperlipidemia/dyslipidemia.

^#^FE: First entry time.

^&^*p*<0·05.

^@^Lp(a): The variable was the log2 transform of Lp(a) level.

**Table 2. Baseline characteristics of patients with statin use and controls of LDL-C Stable Cohort^$^.**

| **Characteristic** | **Item** | **Non-statin use(n=18483)** | **Statin use(n=19062)** |
| --- | --- | --- | --- |
| Age^&^ | <45 | 4537(24.55%) | 1155(6.06%) |
|  | 46-65 | 8423(45.57%) | 7701(40.40%) |
|  | >65 | 5523(29.88%) | 10206(53.54%) |
| Sex | Male | 9348(50.58%) | 9568(50.19%) |
|  | Female | 9135(49.42%) | 9494(49.81%) |
| Comorbidity history^*,&^ | Comorbidity history (FE)^#^ | 2394(12.95%) | 6184(32.44%) |
|  | Non- Comorbidity history (FE) | 16089(87.05%) | 12878(67.56%) |
| Follow-up time^&^ | [0·5 - 3) years | 14504(78.47%) | 12777(67.03%) |
|  | [3 - 5) years | 3117(16.86%) | 4502(23.62%) |
|  | ≥ 5 years | 862(4.66%) | 1783(9.35%) |
| Laboratory results | Lp(a)^@^ at FE (Mean, CI95%, mg/L) ^&^ | 7.90(3.58, 9.87) | 7.82(3.17, 9.87) |
|  | LDL-C (Mean, CI95%, mmol/L) ^&^ | 2.78(1.23, 4.50) | 2.88(1.31, 4.88) |
|  | HDL-C (Mean, CI95%, mmol/L) ^&^ | 1.31(0.69, 2.15) | 1.26(0.73, 2.04) |
|  | APO-A (Mean, CI95%, mmol/L) ^&^ | 1.33(0.76, 1.98) | 1.30(0.81, 1.90) |
|  | APO-B (Mean, CI95%, mmol/L) ^&^ | 0.93(0.45, 1.50) | 0.96(0.50, 1.59) |
|  | TC (Mean, CI95%, mmol/L) ^&^ | 4.81(2.75, 6.96) | 4.94(2.90, 7.48) |
|  | TG (Mean, CI95%, mmol/L) ^&^ | 1.52(0.51, 4.30) | 1.73(0.60, 4.89) |
|  | CRP (Mean, CI95%, mmol/L) ^&^ | 13.57(0.15, 84.66) | 12.82(0.19, 77.54) |

^$^In the cohort (LDL-C Stable Cohort), the LDL-C level of patients was not elevated.

^*^Comorbidity history: diabetes/glycuresis, hypertension, arteriosclerosis/vascular sclerosis, besides high blood lipids/hyperlipidemia/dyslipidemia.

^#^FE: First entry time.

^&^*p*<0·05.

^@^Lp(a): The variable was the log2 transform of Lp(a) level.

**Table 3.Baseline characteristics of patients with statin use and controls of Normal LDL-C Cohort^$^.**

| **Characteristic** |  | **Item** | **Non-statin use (n=3799)** | **Statin use (n=3807)** |
| --- | --- | --- | --- | --- |
| Age^&^ |  | <45 | 993(26.14%) | 234(6.15%) |
|  |  | 46-65 | 1456(38.33%) | 1195(31.39%) |
|  |  | >65 | 1350(35.54%) | 2378(62.46%) |
| Sex |  | Male | 2357(62.04%) | 2441(64.12%) |
|  |  | Female | 1442(37.96%) | 1366(35.88%) |
| Comorbidity history^*,&^ |  | Comorbidity history (FE)^#^ | 3349(88.15%) | 2814(73.92%) |
|  |  | Non- Comorbidity history (FE) | 450(11.85%) | 993(26.08%) |
| Follow-up time^&^ |  | [0·5 - 3) years | 3113(81.94%) | 2757(72.42%) |
|  |  | [3 - 5) years | 508(13.37%) | 723(18.99%) |
|  |  | ≥ 5 years | 178(4.69%) | 327(8.59%) |
| Laboratory results |  | Lp(a)^@^ at FE (Mean, CI95%, mg/L) | 7.26(0.00, 9.32) | 7.24(1.00, 9.42) |
|  |  | LDL-C (Mean, CI95%, mmol/L) ^&^ | 1.41(0.46, 1.79) | 1.47(0.80, 1.79) |
|  |  | HDL-C (Mean, CI95%, mmol/L) | 1.13(0.31, 2.06) | 1.14(0.57, 2.00) |
|  |  | APO-A (Mean, CI95%, mmol/L) ^&^ | 1.14(0.38, 1.88) | 1.19(0.68, 1.80) |
|  |  | APO-B (Mean, CI95%, mmol/L) ^&^ | 0.58(0.23, 0.98) | 0.62(0.35, 0.96) |
|  |  | TC (Mean, CI95%, mmol/L) ^&^ | 3.29(1.54, 5.14) | 3.45(2.26, 5.40) |
|  |  | TG (Mean, CI95%, mmol/L) ^&^ | 1.55(0.33, 7.00) | 1.81(0.49, 7.61) |
|  |  | CRP (Mean, CI95%, mmol/L) ^&^ | 19.11(0.18, 99.12) | 16.54(0.20, 95.33) |

^$^In the cohort (Normal LDL-C Cohort), the LDL-C level of patients was not more than 1·8mmol/L.

^*^Comorbidity history: diabetes/glycuresis, hypertension, arteriosclerosis/vascular sclerosis, except high blood lipids/hyperlipidemia/dyslipidemia.

^#^FE: First entry time.

^&^*p*<0·05.

^@^Lp(a): The variable was the log2 transform of Lp(a) level

**Table 4. Association between treatment with statin-based drugs versus non-statin use and the change of the Lp(a).**

| **Item** | **Non-statin use** | **Statin use** | **HR(CI95%)** | **HR(CI95%)^*^** |
| --- | --- | --- | --- | --- |
| Primary outcome^%^ | Non-statin use (n=35753) | Statin use (n=35572) |  |  |
| Lp(a) decrease (FU) | 14991(41.93%) | 4839(13.60%) | 1.20(1.18,1.22)^&^ | 1.17(1.15,1.19)^&^ |
| Lp(a) increase (FU)) | 20762(58.07%) | 30733(86.40%) |  |  |
| Primary outcome^$^ | Non-statin use(n=18483) | Statin use(n=19062) |  |  |
| Lp(a) decrease (FU) | 7658(41.43%) | 2287(12.00%) | 1.12(1.09,1.15)^&^ | 1.14(1.11,1.17)^&^ |
| Lp(a) increase (FU) | 10825(58.57%) | 16775(88.00%) |  |  |
| Primary outcome^@^ | Non-statin use (n=3799) | Statin use (n=3807) |  |  |
| Lp(a) decrease (FU) | 392(39·36%) | 90(9·04%) | 1.18(1.12,1.24)^&^ | 1.21(1.14,1.28)^&^ |
| Lp(a) increase (FU) | 604(60·64%) | 906(90·96%) |  |  |

^*^Adjusted by Age, CRP, Follow-up(months), LDL-C, Comorbidity (FE), HDL-C, APO-A, APO-B ,and the change of LDL-C.

^%^All Study Cohort: In the dataset, we included all patients who used statin and the baseline balanced counterpart.

^$^LDL-C Stable Cohort: In the dataset, the LDL-C level of patients did not ascend. FU: Follow up.

^@^Normal LDL-C Cohort: In the dataset, the LDL-C level of patients were not more than 1·8mmol/L. FU: Follow up.

^&^*p*<0·05.
