# Supplementary material for: Association of statin use and increase in lipoprotein(a): a real-world database research
Source: Eur J Med Res. 2023 Jul 1;28:212. doi: 10.1186/s40001-023-01155-x (PMC10314451; doi:10.1186/s40001-023-01155-x)
Supplement: Supplementary file 3 — Additional file 3: Description of other three cohorts. [file 40001_2023_1155_MOESM3_ESM.docx]

# Description of Other Three Cohorts

We constructed three different cohorts to evaluate the robustness of the results from Suvalue database. The All Study Cohort included all patients who used statin and their baseline-balanced counterparts, regardless of LDL-C level. LDL-C Stable Cohort in which the LDL-C level of patients with statin use was not elevated over the follow-up time; and the Normal LDL-C Cohort in which the LDL-C level of patients at the first study entry were less than 1·8mmol/L. The All Study Cohort, including all patients regardless of LDL-C levels and composed of patients who used statin and their baseline-balanced counterparts, had 71,325 patients (**Figure 1**); LDL-C Stable Cohort, in which besides their LDL-C level ≥ 1·8mmol / L, the LDL-C level of patients with statin use was not elevated over the follow-up time, including 37,545 patients (**Figure 2**); and the Normal LDL-C Cohort, in which statin-treated patients at the first study entry had the LDL-C level < 1·8mmol/L, had 7,606 patients (**Figure 3**). The demograph of the three cohorts are described (**Table 1-3**).

The positive association between statin use and evaluated Lp(a) was also observed by the three cohorts, the All Study Cohort, the LDL-C Stable, and the Normal LDL-C Cohort. The adjusted hazard ratios and their 95% confidence intervals of the All Study Cohort, the LDL-C Stable Cohort, and the Normal LDL-C Cohort were 1.17 (95% Confidential Interval: 1·15 to 1·19), 1·14(95% Confidential Interval: 1·11 to 1·17), and 1·21 (95% Confidential Interval: 1·14 to 1·28), respectively (**Table 4**).


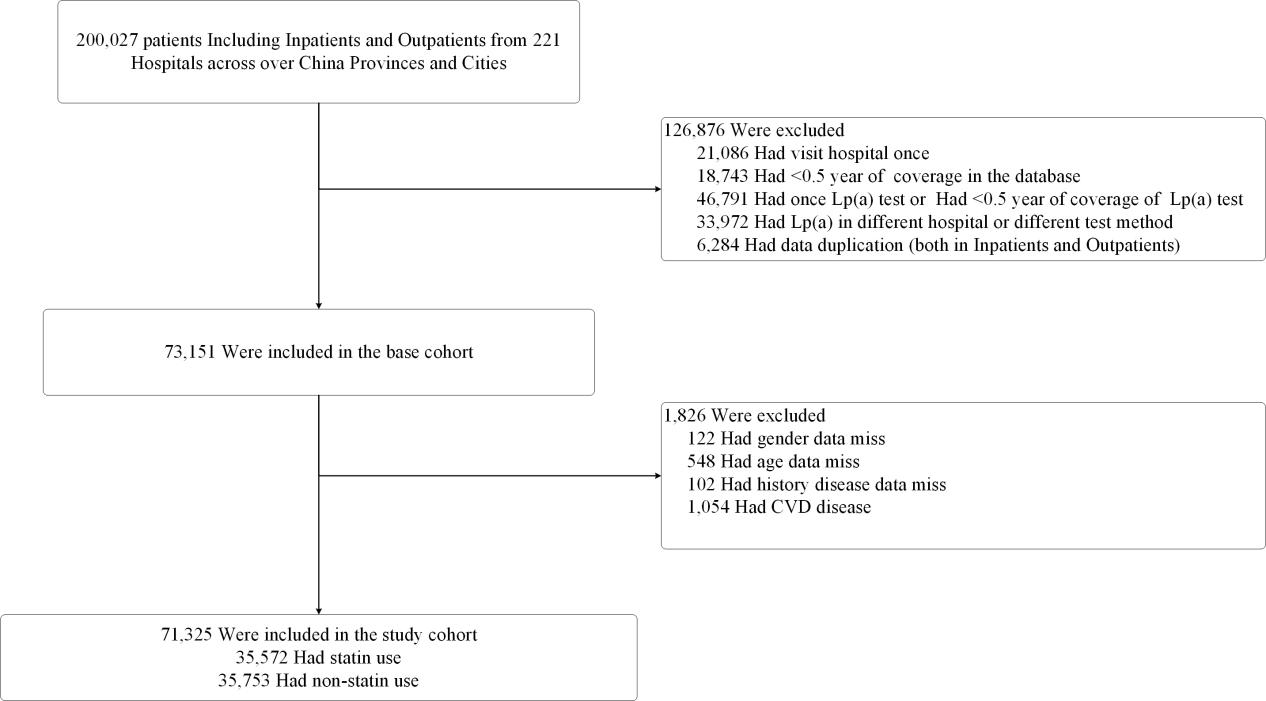


**Figure 1. Numbers of patients in the All Study Cohort.**


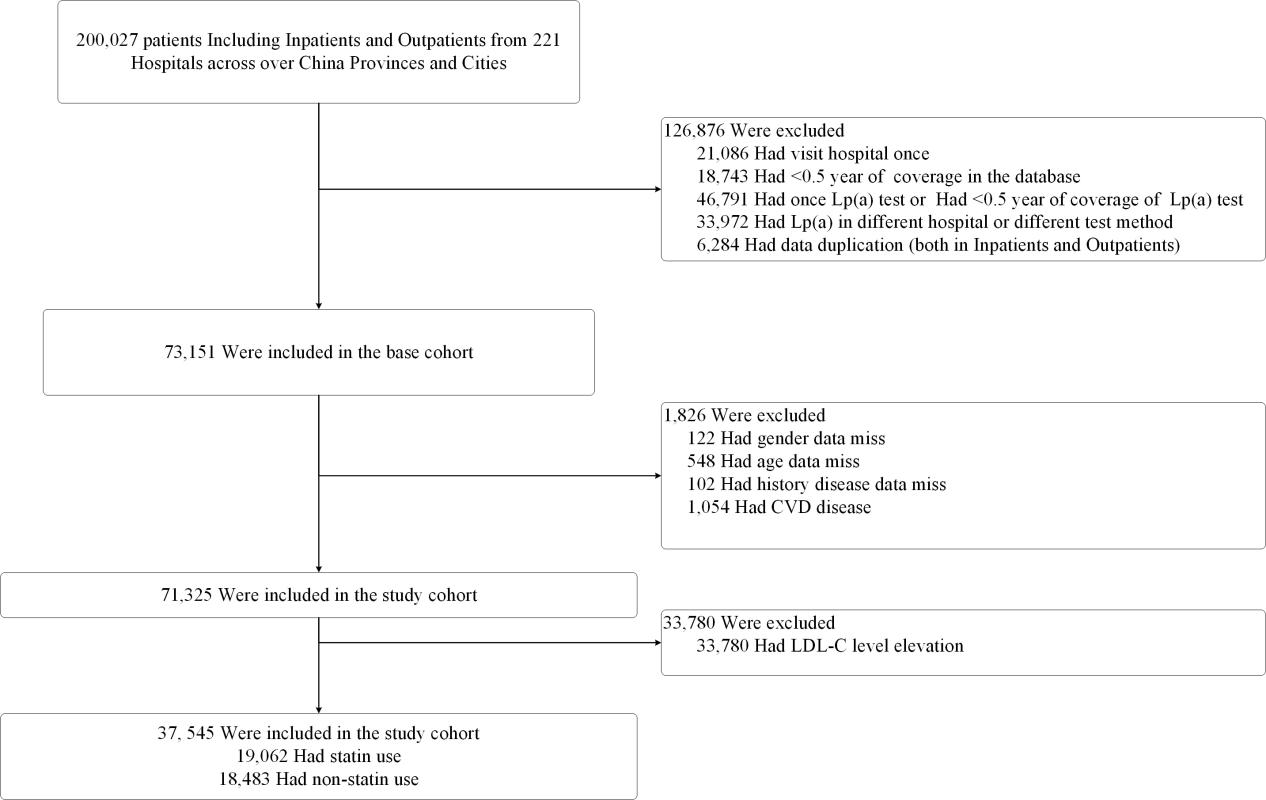


**Figure 2. Numbers of patients in the LDL-C Stable Cohort.**

**
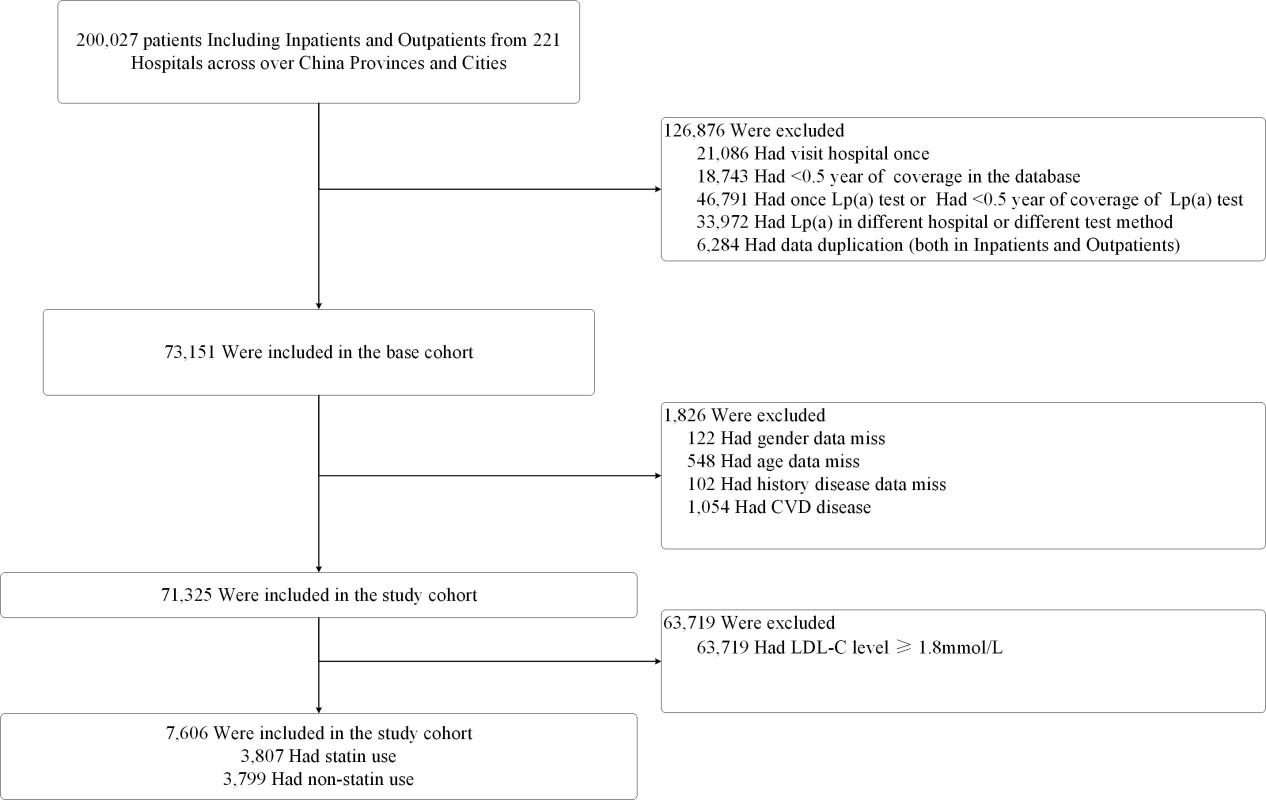
**

**Figure 3. Numbers of patients in the Normal LDL-C Cohort.**

**Table 1. Baseline characteristics of patients with statin use and controls of All Study Cohort ^$^.**

| **Characteristic** | **Item** | **Non-statin use (n=35753)** | **Statin use (n=35572)** |
| --- | --- | --- | --- |
| Age^&^ | <45 | 9492(26.55%) | 2707(7.61%) |
|  | 46-65 | 15917(44.52%) | 14306(40.22%) |
|  | >65 | 10344(28.93%) | 18559(52.17%) |
| Sex^&^ | Male | 18394(51.45%) | 17948(50.46%) |
|  | Female | 17359(48.55%) | 17624(49.54%) |
| Comorbidity history^*,&^ | Comorbidity history (FE)^#^ | 30870(86.34%) | 23892(67.17%) |
|  | Non- Comorbidity history (FE) | 4883(13.66%) | 11680(32.83%) |
| Follow-up time^&^ | [0·5 - 3) years | 27583(77.15%) | 23943(67.31%) |
|  | [3 - 5) years | 6129(17.14%) | 8192(23.03%) |
|  | ≥ 5 years | 2041(5.71%) | 3437(9.66%) |
| Laboratory results | Lp(a)^@^ at FE (Mean, CI95%, mg/L) ^&^ | 7.88(3.58, 9.87) | 7.81(3.19, 9.85) |
|  | LDL-C (Mean, CI95%, mmol/L) ^&^ | 2.78(1.24, 4.50) | 2.89(1.30, 4.87) |
|  | HDL-C (Mean, CI95%, mmol/L) ^&^ | 1.32(0.68, 2.14) | 1.27(0.72, 2.05) |
|  | APO-A (Mean, CI95%, mmol/L) ^&^ | 1.34(0.76, 2.00) | 1.31(0.81, 1.94) |
|  | APO-B (Mean, CI95%, mmol/L) ^&^ | 0.93(0.46, 1.50) | 0.96(0.50, 1.60) |
|  | TC (Mean, CI95%, mmol/L) ^&^ | 4.82(2.75, 6.99) | 4.97(2.91, 7.48) |
|  | TG (Mean, CI95%, mmol/L) ^&^ | 1.54(0.52, 4.41) | 1.77(0.60, 5.10) |
|  | CRP (Mean, CI95%, mmol/L) ^&^ | 13.49(0.17, 81.67) | 12.97(0.18, 76.93) |

^$^In the cohort (All Study Cohort), all patients and the counterparts were included.

^*^Comorbidity history: diabetes/glycuresis, hypertension, arteriosclerosis/vascular sclerosis, besides high blood lipids/hyperlipidemia/dyslipidemia.

^#^FE: First entry time.

^&^*P*<0·05.

^@^Lp(a): The variable was the log2 transform of Lp(a) level.

**Table 2. Baseline characteristics of patients with statin use and controls of LDL-C Stable Cohort^$^.**

| **Characteristic** | **Item** | **Non-statin use(n=18483)** | **Statin use(n=19062)** |
| --- | --- | --- | --- |
| Age^&^ | <45 | 4537(24.55%) | 1155(6.06%) |
|  | 46-65 | 8423(45.57%) | 7701(40.40%) |
|  | >65 | 5523(29.88%) | 10206(53.54%) |
| Sex | Male | 9348(50.58%) | 9568(50.19%) |
|  | Female | 9135(49.42%) | 9494(49.81%) |
| Comorbidity history^*,&^ | Comorbidity history (FE)^#^ | 2394(12.95%) | 6184(32.44%) |
|  | Non- Comorbidity history (FE) | 16089(87.05%) | 12878(67.56%) |
| Follow-up time^&^ | [0·5 - 3) years | 14504(78.47%) | 12777(67.03%) |
|  | [3 - 5) years | 3117(16.86%) | 4502(23.62%) |
|  | ≥ 5 years | 862(4.66%) | 1783(9.35%) |
| Laboratory results | Lp(a)^@^ at FE (Mean, CI95%, mg/L) ^&^ | 7.90(3.58, 9.87) | 7.82(3.17, 9.87) |
|  | LDL-C (Mean, CI95%, mmol/L) ^&^ | 2.78(1.23, 4.50) | 2.88(1.31, 4.88) |
|  | HDL-C (Mean, CI95%, mmol/L) ^&^ | 1.31(0.69, 2.15) | 1.26(0.73, 2.04) |
|  | APO-A (Mean, CI95%, mmol/L) ^&^ | 1.33(0.76, 1.98) | 1.30(0.81, 1.90) |
|  | APO-B (Mean, CI95%, mmol/L) ^&^ | 0.93(0.45, 1.50) | 0.96(0.50, 1.59) |
|  | TC (Mean, CI95%, mmol/L) ^&^ | 4.81(2.75, 6.96) | 4.94(2.90, 7.48) |
|  | TG (Mean, CI95%, mmol/L) ^&^ | 1.52(0.51, 4.30) | 1.73(0.60, 4.89) |
|  | CRP (Mean, CI95%, mmol/L) ^&^ | 13.57(0.15, 84.66) | 12.82(0.19, 77.54) |

^$^In the cohort (LDL-C Stable Cohort), the LDL-C level of patients was not elevated.

^*^Comorbidity history: diabetes/glycuresis, hypertension, arteriosclerosis/vascular sclerosis, besides high blood lipids/hyperlipidemia/dyslipidemia.

^#^FE: First entry time.

^&^*P*<0·05.

^@^Lp(a): The variable was the log2 transform of Lp(a) level.

**Table 3.Baseline characteristics of patients with statin use and controls of Normal LDL-C Cohort^$^.**

| **Characteristic** |  | **Item** | **Non-statin use (n=3799)** | **Statin use (n=3807)** |
| --- | --- | --- | --- | --- |
| Age^&^ |  | <45 | 993(26.14%) | 234(6.15%) |
|  |  | 46-65 | 1456(38.33%) | 1195(31.39%) |
|  |  | >65 | 1350(35.54%) | 2378(62.46%) |
| Sex |  | Male | 2357(62.04%) | 2441(64.12%) |
|  |  | Female | 1442(37.96%) | 1366(35.88%) |
| Comorbidity history^*,&^ |  | Comorbidity history (FE)^#^ | 3349(88.15%) | 2814(73.92%) |
|  |  | Non- Comorbidity history (FE) | 450(11.85%) | 993(26.08%) |
| Follow-up time^&^ |  | [0·5 - 3) years | 3113(81.94%) | 2757(72.42%) |
|  |  | [3 - 5) years | 508(13.37%) | 723(18.99%) |
|  |  | ≥ 5 years | 178(4.69%) | 327(8.59%) |
| Laboratory results |  | Lp(a)^@^ at FE (Mean, CI95%, mg/L) | 7.26(0.00, 9.32) | 7.24(1.00, 9.42) |
|  |  | LDL-C (Mean, CI95%, mmol/L) ^&^ | 1.41(0.46, 1.79) | 1.47(0.80, 1.79) |
|  |  | HDL-C (Mean, CI95%, mmol/L) | 1.13(0.31, 2.06) | 1.14(0.57, 2.00) |
|  |  | APO-A (Mean, CI95%, mmol/L) ^&^ | 1.14(0.38, 1.88) | 1.19(0.68, 1.80) |
|  |  | APO-B (Mean, CI95%, mmol/L) ^&^ | 0.58(0.23, 0.98) | 0.62(0.35, 0.96) |
|  |  | TC (Mean, CI95%, mmol/L) ^&^ | 3.29(1.54, 5.14) | 3.45(2.26, 5.40) |
|  |  | TG (Mean, CI95%, mmol/L) ^&^ | 1.55(0.33, 7.00) | 1.81(0.49, 7.61) |
|  |  | CRP (Mean, CI95%, mmol/L) ^&^ | 19.11(0.18, 99.12) | 16.54(0.20, 95.33) |

^$^In the cohort (Normal LDL-C Cohort), the LDL-C level of patients was not more than 1·8mmol/L.

^*^Comorbidity history: diabetes/glycuresis, hypertension, arteriosclerosis/vascular sclerosis, except high blood lipids/hyperlipidemia/dyslipidemia.

^#^FE: First entry time.

^&^*P*<0·05.

^@^Lp(a): The variable was the log2 transform of Lp(a) level.

**Table 4. Association between treatment with statin-based drugs versus non-statin use and the change of the Lp(a).**

| **Item** | **Non-statin use** | **Statin use** | **HR(CI95%)** | **HR(CI95%)^*^** |
| --- | --- | --- | --- | --- |
| Primary outcome^%^ | Non-statin use (n=35753) | Statin use (n=35572) |  |  |
| Lp(a) decrease (FU) | 14991(41.93%) | 4839(13.60%) | 1.20(1.18,1.22)^&^ | 1.17(1.15,1.19)^&^ |
| Lp(a) increase (FU)) | 20762(58.07%) | 30733(86.40%) |  |  |
| Primary outcome^$^ | Non-statin use(n=18483) | Statin use(n=19062) |  |  |
| Lp(a) decrease (FU) | 7658(41.43%) | 2287(12.00%) | 1.12(1.09,1.15)^&^ | 1.14(1.11,1.17)^&^ |
| Lp(a) increase (FU) | 10825(58.57%) | 16775(88.00%) |  |  |
| Primary outcome^@^ | Non-statin use (n=3799) | Statin use (n=3807) |  |  |
| Lp(a) decrease (FU) | 392(39·36%) | 90(9·04%) | 1.18(1.12,1.24)^&^ | 1.21(1.14,1.28)^&^ |
| Lp(a) increase (FU) | 604(60·64%) | 906(90·96%) |  |  |

^*^Adjusted by Age, CRP, Follow-up(months), LDL-C, Comorbidity (FE), HDL-C, APO-A, APO-B ,and the change of LDL-C.

^%^All Study Cohort: In the dataset, we included all patients who used statin and the baseline balanced counterpart.

^$^LDL-C Stable Cohort: In the dataset, the LDL-C level of patients did not ascend. FU: Follow up.

^@^Normal LDL-C Cohort: In the dataset, the LDL-C level of patients were not more than 1·8mmol/L. FU: Follow up.

^&^*P*<0·05.
